# Individual differences in mental imagery modulate effective connectivity of scene-selective regions during resting state

**DOI:** 10.1007/s00429-022-02475-0

**Published:** 2022-03-21

**Authors:** Maria Giulia Tullo, Hannes Almgren, Frederik Van de Steen, Valentina Sulpizio, Daniele Marinazzo, Gaspare Galati

**Affiliations:** 1 Department of Translational and Precision Medicine, “Sapienza” University of Rome, Via Benevento, 6, 00161 Roma, RM Italy; 2 Brain Imaging Laboratory, Department of Psychology, “Sapienza” University of Rome, Rome, Italy; 3 Cognitive and Motor Rehabilitation and Neuroimaging Unit, IRCCS Santa Lucia Foundation, Rome, Italy; 4 Department of Data Analysis, Faculty of Psychology and Educational Sciences, Ghent University, Ghent, Belgium; 5 Department of Clinical Neurosciences, Cumming School of Medicine, University of Calgary, Calgary, AB Canada; 6 Hotchkiss Brain Institute, University of Calgary, Calgary, AB Canada; 7 AIMS, Center For Neurosciences, Vrije Universiteit Brussel, Brussel, Belgium; 8 PhD Program in Behavioral Neuroscience, “Sapienza” University of Rome, Rome, Italy

**Keywords:** Scene-selective regions, Navigation, Imagery, fMRI, Resting-state, Dynamic Causal Modelling

## Abstract

Successful navigation relies on the ability to identify, perceive, and correctly process the spatial structure of a scene. It is well known that visual mental imagery plays a crucial role in navigation. Indeed, cortical regions encoding navigationally relevant information are also active during mental imagery of navigational scenes. However, it remains unknown whether their intrinsic activity and connectivity reflect the individuals’ ability to imagine a scene. Here, we primarily investigated the intrinsic causal interactions among scene-selective brain regions such as Parahipoccampal Place Area (PPA), Retrosplenial Complex, and Occipital Place Area (OPA) using Dynamic Causal Modelling for resting-state functional magnetic resonance data. Second, we tested whether resting-state effective connectivity parameters among scene-selective regions could reflect individual differences in mental imagery in our sample, as assessed by the self-reported Vividness of Visual Imagery Questionnaire. We found an inhibitory influence of occipito-medial on temporal regions, and an excitatory influence of more anterior on more medial and posterior brain regions. Moreover, we found that a key role in imagery is played by the connection strength from OPA to PPA, especially in the left hemisphere, since the influence of the signal between these scene-selective regions positively correlated with good mental imagery ability. Our investigation contributes to the understanding of the complexity of the causal interaction among brain regions involved in navigation and provides new insight in understanding how an essential ability, such as mental imagery, can be explained by the intrinsic fluctuation of brain signal.

**Supplementary Information:**

The online version contains supplementary material available at 10.1007/s00429-022-02475-0.

## Introduction

To explore the surrounding environment, our brain must be able to recognize, memorize and recall specific places in the world. More specifically, forming and memorizing a mental image (i.e., a cognitive map) of a specific place plays a crucial role in our ability to navigate the spatial world. For this reason, it is plausible that being a good imager might enhance the skills to navigate in the environment. Over the past two decades, some important issues have been clarified regarding the link between imagery and navigational performance. Recently, functional resonance imaging (fMRI) studies clarified that voluntary mental imagery activates several brain regions spanning from frontal areas, such as the dorsal and ventral prefrontal cortex, to medial and temporal areas such as the hippocampus and category-selective regions (Mechelli 2004; Ranganath and D’Esposito 2005; Pearson and Westbrook 2015). A top-down model has been introduced and proposes that mental images are organized and controlled by frontal regions including the inferior frontal gyrus and anterior cingulate cortex (Fulford et al. 2018), information stored in memory is retrieved in medial temporal areas such as the hippocampus, and the content-specific representation of the mental images activates regions in the most posterior part of the brain (Dijkstra et al. 2017). Indeed, the specific content of a mental image activates different brain regions, similarly to what happens during visual perception (Ganis et al. 2004). For instance, imagining a face leads to activation of the fusiform face area (FFA), a brain area activated during face perception; similarly, imaging a scene leads to activation of ventromedial posterior cortical regions (scene-selective regions), which are known to be activated during scene and landscape perception (Epstein and Baker 2019).

The scene-selective regions include at least three visual cortical areas responding selectively to scenes, compared to other category images such as faces and objects. Specifically, these regions are termed the Parahippocampal Place Area (PPA), the Retrosplenial Complex (RSC), and the Occipital Place Area (OPA), which are located ventrally, medially, and posteriorly on the cortical surface, respectively. These regions play different and complementary roles, and several neuroimaging studies demonstrated the contribution of each aforementioned brain region in scene processing. PPA, located at the boundary between the posterior parahippocampal cortex and the anterior lingual gyrus, is activated by passive viewing of real-world scenes or landmarks (Epstein and Kanwisher, 1998); The Retrosplenial complex extends from the retrosplenial cortex itself, located posteriorly to the corpus callosum, through the posterior cingulate cortex to the anterior bank of the parietal-occipital fissure (Burles et al. 2017). RSC is mostly active during both real and imagined navigation (Maguire 2001; Ino et al. 2002; Wolbers 2005), retrieval of environment-centered information (Committeri et al. 2004; Galati et al. 2010), and is selectively sensitive to a change in point of view (Sulpizio et al. 2016), mental imagery of familiar places (Boccia et al. 2015) and encoding of permanent items (Auger et al. 2012; Auger and Maguire 2013). More recently, a growing number of studies have disclosed the role of OPA in scene perception. OPA, which is located around the transverse occipital sulcus is retinotopically organized (Nasr et al. 2011) and shows a preference for the lower visual field (Silson et al. 2015), encodes environmental boundaries (Julian et al. 2016) and local navigational affordances (Bonner and Epstein 2017), and represents motion information in the immediately visible scene from a first-person perspective (Kamps et al. 2016). These results suggest that OPA is specialized in encoding both low- and high-level characteristics such as environmental affordances of a scene (Epstein and Baker 2019) and might be a primary site toward higher cortical regions.

Several neuroimaging human studies revealed that PPA, RSC, and OPA are strongly interconnected. Studying functional connectivity among scene-selective and hippocampal regions at rest, Boccia et al. (2017a) found that PPA is connected to occipito-temporal areas including RSC, lingual gyrus, the calcarine cortex, the parieto-occipital sulcus, and the posterior hippocampus. Similarly, Silson et al. (2015) found that OPA is significantly more connected to the posterior portion of the parahippocampal area (pPPA) than to the anterior portion of the parahippocampal area (aPPA) while the retrosplenial complex/medial parietal cortex (MPA) is more strongly connected with aPPA than pPPA. Similar results were obtained by Baldassano et al. (2013)

In summary, PPA, RSC, and OPA are key nodes of a network supporting both perception and imagery of environmental scenes. Although the functional coupling between these regions seems to be well established in humans based on previous resting-state functional imaging studies (Margulies et al. 2009; Nasr et al. 2013), information about the directed causal interactions among PPA, RSC, and OPA is still lacking. Here, we used spectral Dynamic Causal Modelling (DCM) (Friston et al. 2003, 2014) for resting-state fMRI to assess the strength of the influence that these regions exert on each other without any explicit task. After assessing the dynamic functional connectivity in terms of directed effective connectivity among scene-selective regions, we also tested whether individual differences in connectivity estimates could be related to individual differences in mental imagery ability.

Using resting-state functional connectivity, we previously found that the pattern of reciprocal connections between scene-selective regions reflects individual differences in spatial navigation (Sulpizio et al. 2016; Tullo et al. 2018). In particular, the vividness of visual imagery seems to have a key role in a wide range of cognitive abilities (Pearson 2019), including successful performance on mental rotation (Pazzaglia and Moè 2013), spatial tasks (Piccardi et al. 2017), and navigation (Marchette et al. 2011; Keogh and Pearson 2011, 2014). “Seeing with the mind’s eye”, i.e., imaging something, assumes that visual information arises from memory or likewise can be combined and modified to make a strategy or to achieve a goal (Kosslyn et al. 2001). In this sense, having good imagery abilities plays a crucial role in constructing and recalling a schematic representation of the environment (e.g., a map), adapting own position to the surrounding place, and consequently getting the easiest way-finding. In this framework, several studies revealed that individual differences in cognitive style such as visual or verbal strategy (Blazhenkova and Kozhevnikov 2009, 2010) affect the type of information individuals choose to guide navigation (Pazzaglia and Moè 2013; Kraemer et al. 2017; Piccardi et al. 2017). Indeed, subjects who select and search for salient landmarks rely on a *landmark strategy*; subjects who learn the path using egocentric coordinates (for example, where to turn, right or left) rely on a *route strategy*; subjects who use both egocentric and allocentric coordinates rely on a *survey strategy* and they have a clearer global map of the surrounding environment (Pazzaglia and De Beni 2001). It has been found that survey individuals re-orient themselves faster than landmarks and route-individuals because they are more independent from the space around them. Mental imagery is at the base of this ability. It has been found that a preference for verbalizing descriptions is useful for retrieving position information but not for giving the relative spatial location, while a visual cognitive strategy is useful for judging relative direction among landmarks (Nori and Giusberti 2006; Kraemer et al. 2017).

Here, we used DCM applied to rs-fMRI and a self-report questionnaire on mental imagery skills, which assess the vividness of visual mental images (Vividness of Visual Imagery Questionnaire, VVIQ) (Marks, 1973), to test the hypothesis that individual differences in mental imagery abilities account for how scene-selective regions interact with each other. Results of this study point out that causal information flow among scene-selective regions is relevant to understand individual differences, in particular, mental imagery ability that plays a crucial role in successful navigation in the surrounding environment.

## Method

### Participants

A total of 42 healthy volunteers (mean age = 32.21, SD = 4.17, 24 female) with normal or corrected vision participated in this study. Each subject underwent two fMRI sessions including two localizer scans for scene-selective regions and two scans of resting-state. Each scan began less than a minute after the other unless the subject asked for a brief break inside the scanner. Magnetic resonance imaging (MRI) data were collected on a Siemens Allegra 3T at the Santa Lucia Foundation in Rome (Italy) for 18 of the 42 participants and on a Philips Achieva 3T scanner at the Institute for Advanced Biomedical Technologies (ITAB) of the University G. D’Annunzio Foundation in Chieti (Italy) for the remaining 24 subjects. All participants were right-handed, as assessed by the Edinburgh Handedness Inventory (Oldfield 1971) and had a normal or corrected-to-normal vision. All volunteers gave their written informed consent to participate in the study that was previously approved by the research ethics committees either at Fondazione Santa Lucia or at University G. D’Annunzio, according to the Declaration of Helsinki.

### Experimental paradigm

The localizer fMRI experiment consisted of 8 blocks (16 s) of passive viewing of faces alternated with 8 blocks (16 s) of passive view of navigationally relevant stationary stimuli such as places, with each image presented for 300 ms every 500 ms, interleaved with fixation periods of 15 s on average. Half of the pictures consisted of common indoor and half of common outdoor scenes. Pictures of faces represented faces with neutral expressions of male (50%) and female (50%) young adults. In the resting-state fMRI experiments, subjects were laying with eyes closed, they were asked not to think about anything in particular, and no experimental task was imposed.

### Vividness of visual imagery questionnaire

The vividness of visual imagery was measured using the Vividness of Visual Imagery Questionnaire (VVIQ) (Marks 1973). The VVIQ is a self-reported questionnaire composed of 16 items arranged in blocks of four items. Subjects were asked to visualize specific images (e.g., “visualize a raising sun. Carefully consider the following picture that comes in your mind’s eye: a rainbow appears”), and to rate the vividness of each image using a 5-point Likert scale from 1 (clear image such as a real view) to 5 (no image). Twenty-eight of forty-two participants answered to VVIQ questionnaire. We reversed participants’ scores to have low scores for poorer imagery ability and high scores for better imagery ability.

### Image and acquisition processing

Functional T2*-weighted images were collected using a gradient-echo EPI sequence using blood-oxygenation level-dependent (BOLD) contrast over the whole brain (Kwong et al. 1992). BOLD scans collected on Siemens Allegra 3T scanner included thirty contiguous 4-mm slices and were acquired with an in-plane resolution of 3 × 3 mm in an interleaved excitation order (echo time [TE] = 30 ms, repetition time [TR] = 2 s, flip angle = 70°). For localizer and resting-state scans, 242 and 128 volumes were acquired, respectively, resulting in acquisitions lasting about 8′04″ and 4′16″.

BOLD images collected on Philips Achieva 3T scanner included 39 contiguous 4-mm slices, acquired in an interleaved order (voxel size = 3.6 × 3.6 × 3.6 mm, [TE] = 25 ms, [TR] = 1.914 s, flip angle = 80°). For localizer and resting-state scans 249 and 160 volumes were acquired, respectively, resulting in acquisitions lasting about 7′56″ and 5′06″, respectively. Structural images were collected using a sagittal magnetization-prepared rapid acquisition gradient echo (MPRAGE) T1-weighted sequence. Imaging parameters for Allegra MPRAGE scans were as follows: [TE] = 4.4 ms, flip angle = 8°, in-plane resolution = 0.5 × 0.5 mm, slice thickness = 1 mm. Imaging parameters for Achieva MPRAGE scans were as follows: [TE] = 3.7 ms, flip angle = 8°, voxel size = 1 × 1 × 1 mm.

A field map was not part of the acquisition session in both MRI scanners. Although information about the distribution of the static magnetic field is useful to correct for geometric distortions in EPI scans and thus to enhance registration between functional and structural images, and indirectly between participants, in our case, the lack of distortion correction is expected to have a minimal or no impact at all, since the regions of interest considered in the present study were defined on the basis of an analysis of functional data at the individual level (see below).

Preprocessing was performed using the SPM12 software package (Wellcome centre for Human Neuroimaging, London). In each scan, we discarded the first four volumes from data analysis to exclude non-steady-state scans. Resting-state and localizer images were corrected for differences in slice timing, using the central slice of each volume as a reference. Images were realigned to the first functional volume of each session and were coregistered to the skull-stripped anatomical image. Finally, images were normalized to MNI space (Mazziotta et al., 1995) and smoothed using a Gaussian kernel with a 6-mm FWHM. A resting-state effective connectivity analysis between theoretically motivated regions of interest (ROIs) was then performed.

### Region of interest definition

**Fig. 1:**
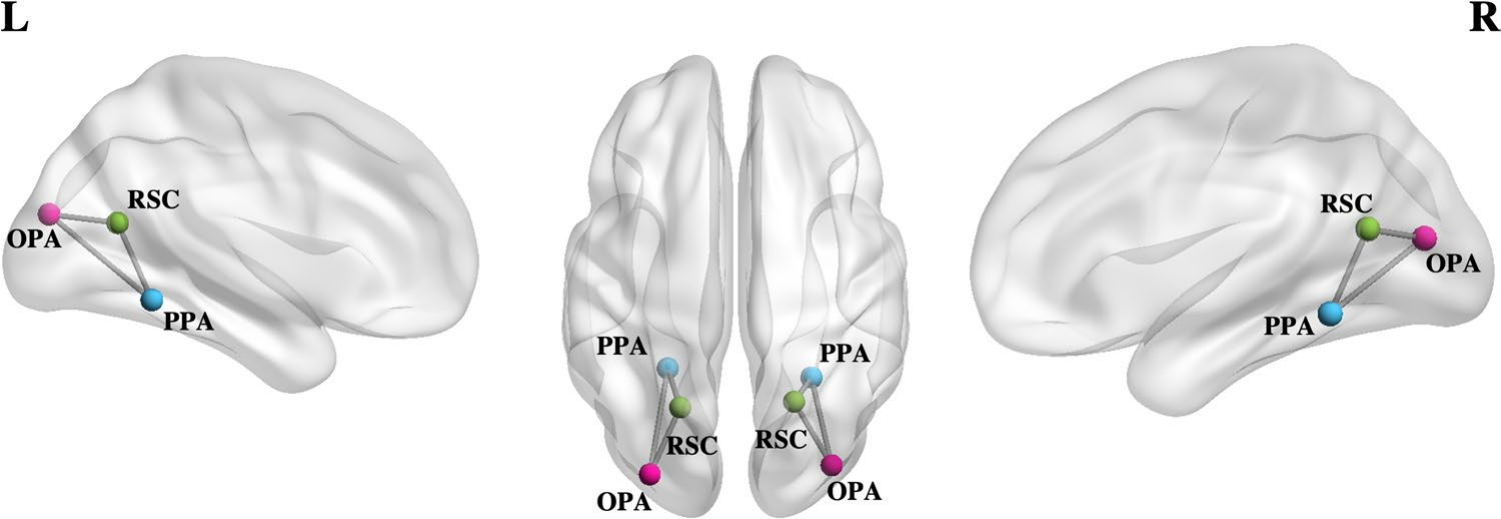
Anatomical location of regions of interest. Nodes of each region are displayed in different colours: the Parahippocampal Place Area (PPA) is represented in sky-blue colour; Retro-splenial complex (RSC) is represented in green and Occipital Place Area (OPA) is shown in pink colour. The edges between regions represent the connections separately modelled for each hemisphere in the Dynamic Causal Modelling (DCM) analysis. Regions of each hemisphere, left (L) and right (R) were visualized using the BrainNet Viewer (Xia et al. 2013)

Regions of interest were defined in volume space on each individual hemisphere separately: Parahippocampal Place Area (PPA), Retrosplenial complex (RSC), and Occipital Place Area (OPA) were defined in each participant by analyzing localizer imaging runs. Place and face blocks were modelled with box-car functions, convolved with a canonical hemodynamic response function. The scene-responsive ROIs were defined as the regions responding more strongly to places than to faces. Each ROI was defined as the conjunction of the results of a T-contrast (places > faces), a sphere (8 mm radius) around the subject-specific local maxima, and a 10 mm sphere radius around ROI centre coordinates defined at the group level. The statistical parametric map resulting was thresholded at *p* < 0.05 FDR corrected at the cluster level, after applying a cluster-forming threshold of *p* < 0.001 uncorrected at the voxel level. The anatomical localization of ROIs is shown in Fig. 1, whereas the mean coordinates and standard deviation of the regional peaks are detailed in Table 1.

**Table 1:** Mean and Standard Deviation of MNI coordinates (X, Y, Z) of the Parahippocampal Place Area (PPA), the Occipital Place Area (OPA) and the Retrosplenial Complex (RSC) in left and right hemispheres

Hemisphere	Regions	MNI Coordinates		
		X	Y	Z
Left	PPA	− 24.5 ± 2.1	− 47.4 ± 2.2	− 11.9 ± 2.3
	OPA	− 31.0 ± 2.1	− 85.7 ± 2.8	16.6 ± 3.3
	RSC	− 20.0 ± 2.2	− 61.5 ± 3	14.0 ± 3.7
Right	PPA	27.4 ± 1.9	− 50.7 ± 3.7	− 10.5 ± 2.6
	OPA	34.7 ± 2.6	− 82.4 ± 2.3	12.1 ± 3.1
	RSC	21.1 ± 1.9	− 59.4 ± 2.7	14.7 ± 3.8

Resting-state data were first modelled with a general linear model (GLM) containing six head motion regressors (three translational, three rotational), cerebrospinal fluid (CSF) signal, white matter (WM) signal, and discrete cosine basis regressors (frequency range 0.0078–0.1 Hz). An F-contrast was specified across all DCT components to produce an SPM. Time-series were extracted by computing the principal eigenvariate of signals from voxels centred on the peak voxel within each ROI and were subsequently used for DCM analyses.

### Dynamic causal modelling (DCM) analysis

We used spectral DCM for resting-state fMRI (Friston et al. 2014) to estimate intrinsic effective connectivity among the scene-selective brain regions from resting-state BOLD responses. Briefly, in the A-matrix, we modelled the strength in units of Hertz (Hz) of the connection among regions. In other words, we observed the rate of change in activity (per second) in one region affected by another. As a constraint of our model, we used a fully connected model, i.e., each region was connected with the other two areas of the same hemisphere (Fig. 1). Each parameter of the DCM A-matrix has a biophysical interpretation since a positive value means that the connection is excitatory (i.e., a region increases the activity in another region); on the contrary, if the parameter has a negative value the connection is inhibitory (Zeidman et al. 2019). In line with what has been done before (Almgren et al. 2018), we specified and inverted DCMs (with all possible connections among regions, i.e., a “full model”) for each session separately without specifying any exogenous input (DCM 12; revision 6801). We excluded from the analysis the sessions that did not meet the following criteria: (1) explained variance of predicted BOLD signals above 60%, (2) at least one connection with a strength greater than 1/8 Hz, and (3) at least one effectively estimated parameter (based on Kullback–Leibler divergence of posterior from prior distribution). These criteria were used as diagnostic tools for detecting so-called ‘bad’ model fits. Since DCM uses a gradient-based approach to maximize the approximate log model evidence, the initial parameter regime might be located in a local plateau. As a consequence, the inversion scheme is ‘stuck’ and hence is unable to obtain a good fit with a valid log model evidence bound.

To assess the relationship between imaginative abilities and connectivity estimates, a multilevel hierarchical linear model using Parametric Empirical Bayes (PEB) was performed (Friston et al. 2016). At the subject level, the average connectivity across sessions was modelled using PEB. Then, two PEB analyses were performed: first, we performed a group-level PEB with a constant term (i.e., the average across subjects) for all the forty-two participants. Secondly, we performed a PEB using both a constant term and VVIQ-scores as regressors for, respectively, modelling the average connectivity across subjects and the individual differences in connectivity in terms of differences in imaging abilities in 28 participants. A posterior probability criterion of 95% for each group-level parameter estimate was used to infer average effective connectivity and the relation with VVIQ. Since we used two datasets, we controlled for the potential confound of using two different scanners (Philips Achieva and Siemens Allegra) to collect data and we performed two PEB analyses modelling the average connectivity across subjects, separately for each scanner. Results revealed comparable results for the two datasets even though some differences in connectivity estimates occurred (see Supplementary Material). Furthermore, limits of analysing data from two datasets are discussed below (see Discussion).

## Results

### Group average connectivity strengths

**Fig. 2:**
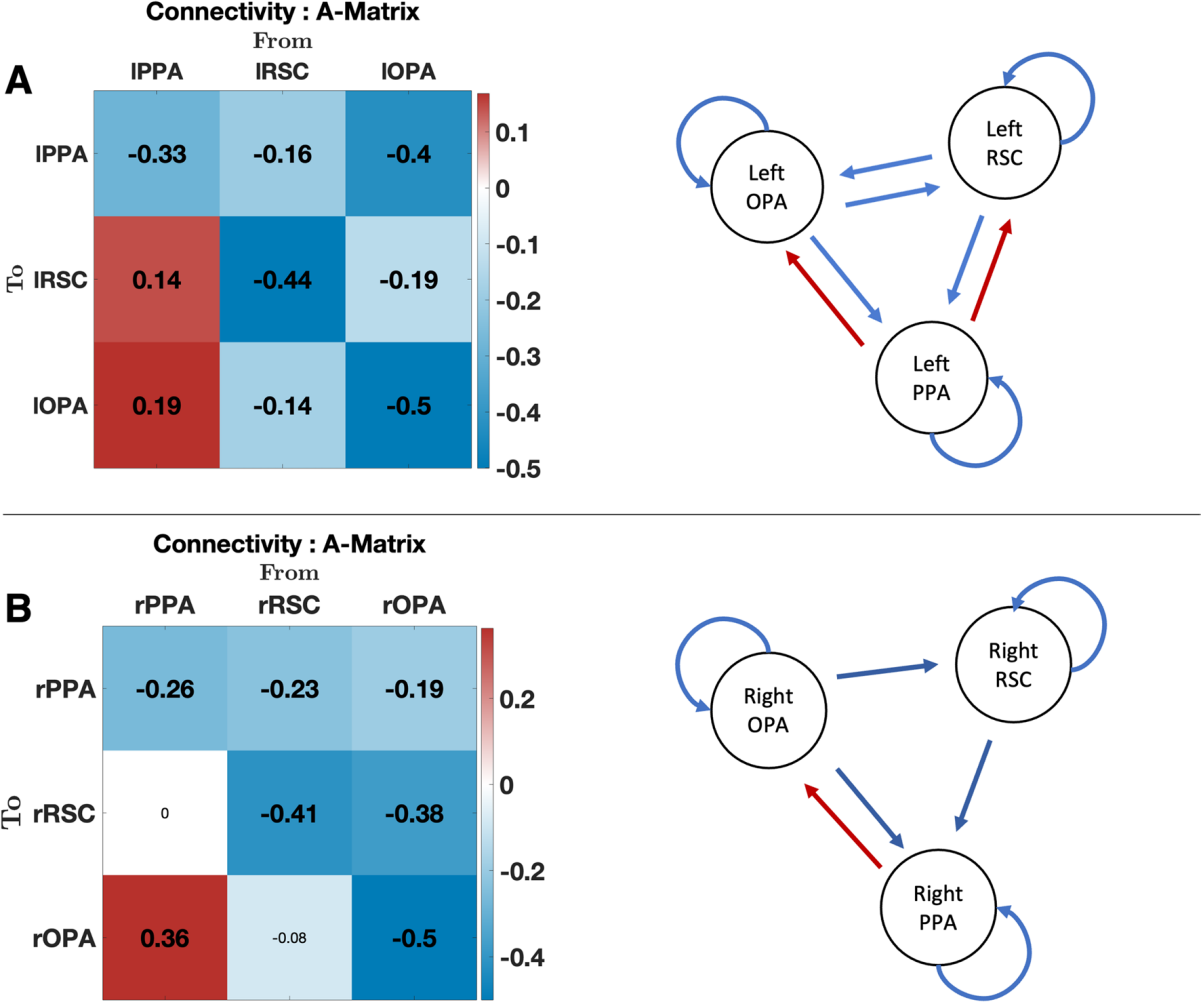
PEB results of intrinsic effective connectivity. A-matrices of intrinsic effective connectivity in the left and right hemisphere are shown in panel A and panel B, respectively. Parameters with posterior probability higher than 95% are shown and are marked in bold type. Non suprathreshold parameters values are also reported in non-bold type in matrices. Connection strengths are displayed from pale red to dark red (i.e., excitatory), and from pale blue to blue (i.e., inhibitory). Schematic representations of A-matrices parameter results are also shown: red solid arrows represent excitatory connections and blue solid arrows represent inhibitory connections. Regions of interest are labelled as follows: Occipital Place Area (OPA), Parahippocampal Place Area (PPA) and Retrosplenial Complex (RSC)

The PEB analysis, modelling the average connectivity on 42 subjects, revealed that the left and the right hemispheres have similar intrinsic connectivity strength at rest (Fig. 2). Indeed, the Retrosplenial complex and the Occipital Place area have an inhibitory role on the other regions in both hemispheres. In the left hemisphere, RSC inhibited PPA (connection strength = − 0.16, posterior probability = 0.99) and OPA (connection strength = − 0.14, pp = 0.99). The left OPA inhibited the left PPA (connection strength = − 0.4, pp = 1.00) and the left RSC (connection strength = -0.19, posterior probability = 0.99). Concurrently, in the right hemisphere, RSC inhibited PPA (connection strength = − 0.23, posterior probability = 1.00) but the influence of right RSC on right OPA didn’t exceed the posterior probability (pp < 0.95). At the same time, the Parahippocampal Place area has an excitatory role onto the other regions more in the left than in the right hemisphere. Indeed, in the left hemisphere, PPA excited both the RSC (connection strength = 0.14, pp = 0.99) and OPA (connection strength = 0.19, pp = 1.00). In the right hemisphere, PPA excited OPA (connection strength = 0.37, pp = 1.00) but the connectivity parameter from right PPA to right RSC did not survive to the posterior probability criterion in the right hemisphere.

### Connectivity strengths and the relationship with VVIQ score

**Fig. 3:**
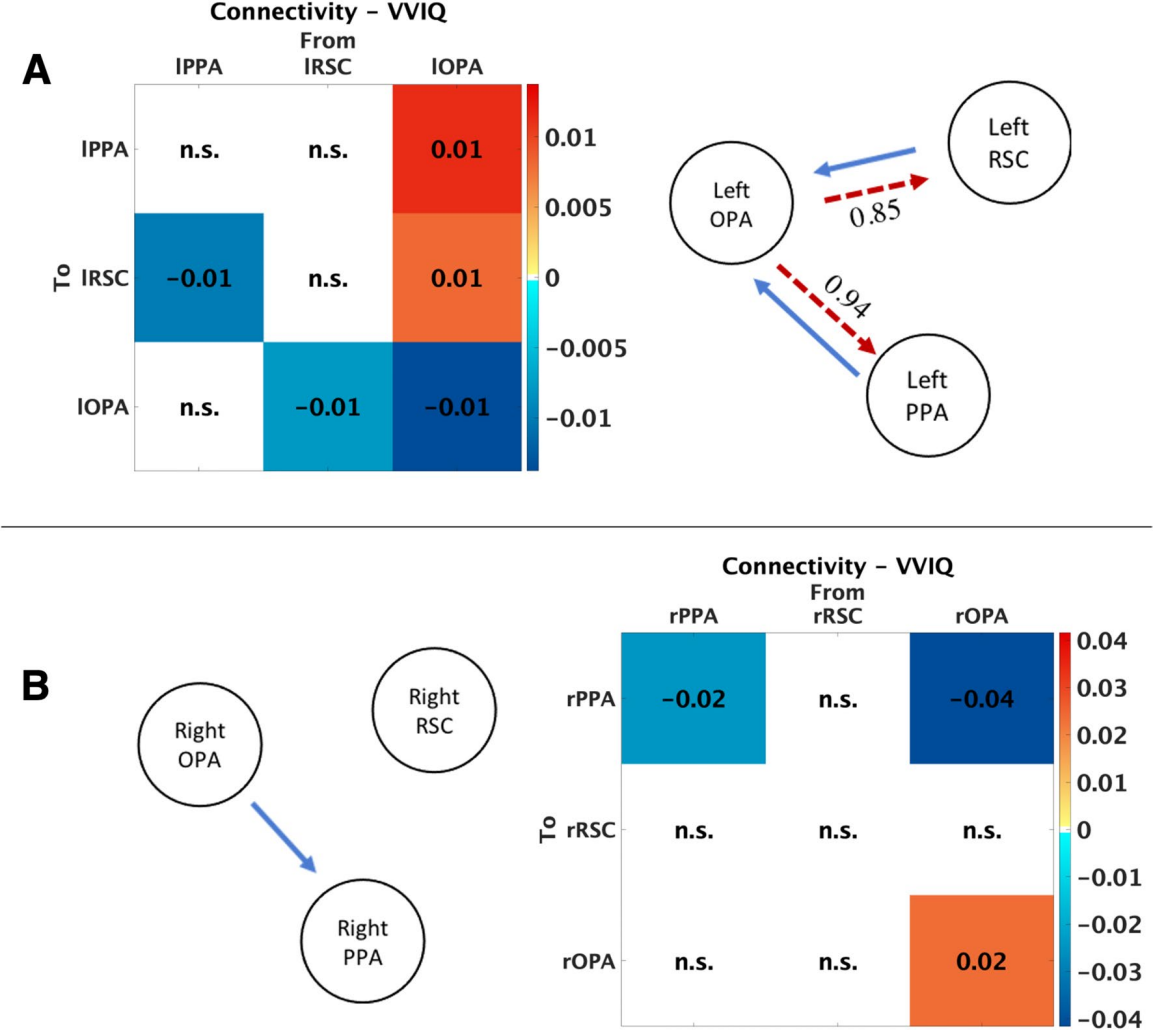
Effect of the Vivideness of Visual Imagery Questionnaire (VVIQ) on each connectivity parameter. Parameters with posterior probability higher than 95% are shown (pp > 0.95) whereas non-suprathreshold parameter values are labelled with “n.s.”. A positive relation between connectivity parameter and VVIQ is shown from yellow to dark red, and a negative relation is shown from turquoise to dark blue. On the right of the panels, a schematic representation for each hemisphere is also provided. Red solid line represents a positive relation between the outgoing connectivity and VVIQ scores whereas the blue solid line represents a negative relation between the outgoing connectivity and VVIQ scores. Dotted lines display parameter values with a posterior probability between 0.85 and 0.95 threshold: Connection strength from left OPA to left PPA is equal to -0.011 (pp = 0.94) and connection strength from left OPA to left RSC is equal to 0.009 (pp = 0.85)

The vividness of Visual Imagery Questionnaire (VVIQ) was used to assess the vividness of mental images in 28 of 42 participants. Lower VVIQ scores reflected poorer imagery abilities and higher scores reflected better imagery abilities. The effect of VVIQ on each connectivity parameter is shown in Fig. 3. Concerning the VVIQ, a different association of the right and left hemispheres with visual imagery was found. In the left hemisphere, a negative association between imagery abilities and the outgoing connectivity from RSC to OPA (connection strength = − 0.008, pp = 0.98) and from PPA to OPA (connection strength = − 0.010, pp = 0.96) was found. On the contrary, the outgoing connectivity from left OPA to both the other scene-selective regions was positively related to good imagery abilities: the outgoing connectivity from OPA to PPA (connection strength = 0.011, pp = 0.94) and from OPA to RSC (connection strength = 0.008, pp = 0.85) was positively associated with VVIQ scores, even if the connectivity parameter between OPA and RSC did not exceed the threshold (pp = 0.85). Conversely, the posterior parameter from left OPA and left PPA (connection strength = 0.011) is equal to 0.94, an acceptable threshold value. In the right hemisphere, the connectivity from OPA to PPA is negatively related to good imagery (parameter = − 0.041, pp = 1.00). Here, we did not consider the relationship between VVIQ and the self-connectivity (right PPA to right PPA = − 0.024, pp = 1.00; right OPA to right OPA = 0.024, pp = 1.00) since we were interested in the modulatory connectivity among scene-selective regions.

## Discussion

In the present study, we primarily aimed at investigating the intrinsic causal interactions between scene-selective regions—the Parahippocampal Place Area (PPA), the Occipital Place Area (OPA) and the Retrosplenial Complex (RSC). Second, in a sub-sample, we tested whether resting-state effective connectivity parameters were associated with mental imagery ability, as assessed by a self-reported questionnaire on vividness imagery. To this aim, we explored the causal influence of three regions involved in scene perception and imagery (PPA, RSC, OPA) in both hemispheres using Dynamic Causal Modelling for resting-state fMRI. The first finding of this study is that both RSC and OPA have an inhibitory role at rest on the other regions and the more ventral region PPA excites the visual area OPA in the right hemisphere, while both OPA and RSC in the left hemisphere show an excitation feedback influence of higher level regions on the lowest ones. It is important to remember that we took into account the spontaneous connectivity among three scene-selective regions. Several studies have focussed on the modular nature of the spontaneous low-frequency fluctuations of the BOLD signal, resulting in the network of areas called intrinsic connectivity networks (Raichle 2011, 2015). Regions such as RSC are part of the “default mode network” (DMN) in which the activity at baseline is always higher than in other brain regions and their functions are never turned off but only enhanced or attenuated (Raichle 2015). Therefore, the “quiet” state of the brain is already ordered to some degree, serving as a potential “scaffold” for underpinning a variety of functional tasks. The information to be memorized, the movement to be learned are configured with existing constraints. In this scenario, people have a certain degree of pre-organization representing a constraint which is not static but can evolve dynamically. In this sense, the intrinsic brain activity and connectivity may reflect the individual skills. Previous studies used resting-state data to correlate individual differences in navigational abilities (Sulpizio et al. 2016) or in different topics such as executive functions (Reineberg et al. 2015) or pain sensitivity (Spisak et al. 2020). According to our knowledge, there are no studies that have explored the effective connectivity among the scene-selective regions at rest. The present results give a first exploratory outlook on how these regions interact with each other. Certainly, results were found to reflect the signal at baseline that could change depending on the task to be performed. In our results, OPA has an inhibitory influence on the other regions at rest. In visual perception, the primary visual areas usually have an excitatory modulation on the higher level regions responding to a specific content of the image. In other tasks, for instance during imagery, the imagery input comes from frontal regions towards the lower level regions (Mechelli 2004; Dijkstra et al. 2017). It is plausible to hypothesize that during a visual perception task the connectivity strength from lower level regions, such as OPA, to higher level ones, such as RSC and PPA, may be excitatory rather than inhibitory during rest. Future studies could fruitfully explore this issue further by examining the effect of individual variability on the brain connectivity also during an active task.

In the present study, we analysed data acquired from two different scanners (Siemens Achieva and Philips Allegra). The use of different scanners could be a potential confound, since different sequence parameters and contextual factors, such as location and hospital personnel, may occur. Despite this, generalizing across multiple sites and acquisition parameters should be as important as generalizing across subjects. Here, to overcome the limitations associated with site differences, we performed two PEB analyses separately for each scanner which revealed comparable results between the scanners even though some differences in connectivity estimates occurred (see Supplementary Materials). Notably, PEB analysis using small sample sizes can be sensitive to differences in subject-specific DCM estimates. In our case, the scanner-specific samples might not have enough subjects to give precise and robust connectivity estimates (e.g., because the priors have too much influence on the estimates). Hence, connections might change because of incomplete sampling and may be underestimated (i.e., because the prior mean is zero). As a result, reporting the average effective connectivity in a large sample size preserves to detect the effect of interest by including only the steady couplings. However, future studies are required to get robust estimates of connectivity among the scene-selective regions by increasing the sample size or by comparing data from different scanners with equivalent—and large—sample size.

To define individual differences in the connectivity pathway among scene-selective regions between good and poor imagers we used the VVIQ score as a covariate in PEB model analysis. We found that the influence of the signal from the left OPA to the ipsilateral PPA is positively related to the VVIQ score. It is well known that the analysis of category-related information takes place in the cortical regions of the ventral visual pathway (VVP) (Epstein and Kanwisher 1998). In this part of the brain, the recognition of higher visual features takes place to distinguish, for instance, a place from a face. Indeed, in the VVP are located both the Parahippocampal Place Area and the Fusiform Face Area (FFA), responding preferentially to faces. Additionally, colour-selective regions, responding to coloured but not to black and white images, are interposed between FFA and PPA (Lafer-Sousa et al. 2016).

Importantly, PPA was found to be involved also during the active learning of landmarks in a spatial navigation task (Aguirre et al. 1996), during the retrieval of spatial information associated with objects previously seen (Janzen and van Turennout 2004), and during the encoding of emotionally relevant cues (Chan et al. 2014), resulting in a more efficient and responsive navigation behaviour. All this evidence supports the idea that PPA is not only involved in the visual perception of static images of places but also during the implicit learning of spatial information. The Vividness of Visual Imagery questionnaire includes items strictly related to the shape of an image coming into the mind’s eye, such as “think of a country scene which involves trees, mountains, and a lake. Consider the picture that comes before your mind’s eye: the contours of the landscape” or “the colour and shape of the lake” (Marks 1973). All the mental images that are asked to recall in the VVIQ questionnaire belong to scenes that subjects know very well such as the sun, trees, flashes of lightning, clouds, mountains. To recall the *contours* of a landscape requires that the elements of that scene are previously recognized, elaborated and stored in memory. It is plausible to hypothesize that the more the visual area OPA, that visually guides the low-level features of scenes, is connected with the higher order visual area PPA, the better the peculiar features of a scene are recognized. In the present study, we shed light on the dynamic couplings between scene-selective regions at rest and we correlated these results with individual differences on vividness on mental imagery. Further studies are required to investigate the vividness of mental imagery during an active task.

The ventral area PPA is known to respond more to scene categorization, distinguishing from an indoor to an outdoor place (e.g., a kitchen from a beach), but not to the change of view in a scene, unlike the more medial area RSC. Here, we found that the outgoing connectivity from OPA to RSC positively correlated with VVIQ in the left hemisphere, but the posterior probability did not exceed the threshold (pp = 0.85). The retrosplenial complex is the region that, more than the other scene-selective regions, is essential for building a “cognitive map” of the surrounding space, elaborating an egocentric view of the scene (Dilks et al. 2011; Persichetti and Dilks 2016) and integrating individual viewpoints into a global representation of the environment. It has a pivotal role in memory encoding and retrieval since it is more active during the retrieval of environmental information of familiar scenes (Epstein et al. 2007). Moreover, RSC was found to be sensitive not only to heading information of perceived environments (Baumann and Mattingley 2010) but also of imagined environments (Vass and Epstein 2016). It should be noted that some items of the VVIQ ask to imagine the scene from a specific perspective: “Think of the front of a shop which you often go to. Consider the picture that comes before your mind’s eye: The overall appearance of the shop from the opposite side of the road” (Marks 1973). Likewise, “You are near the entrance. The colour, shape and details of the door.” However, most of the VVIQ items refer to the shape and contour of a scene instead of viewpoints and this may explain why, in the present study, the outgoing connectivity from OPA to RSC did not exceed the threshold. Further research is needed to assess a positive association from the coupling between OPA and RSC and behavioural measures coming from visuospatial tasks.

Overall, each scene-selective region contributes in a different and complementary way to the categorization, learning and retrieval of environmental information (for a recent and thorough review, see Baumann and Mattingley 2021). As a matter of fact, also neuropsychological studies demonstrate that selective lesions to PPA or RSC cause different impairments in scene elaboration, suggesting that these regions have a complementary role in navigation. Indeed, lesions to PPA seem to be associated with topographical disorientation, in some cases anterograde and retrograde (Takahashi and Kawamura 2002): patients are not able to recognize known and novel scenes and buildings. On the other hand, when RSC is damaged, patients lose their way in familiar or novel environments with an inability to access their cognitive map.

Individual differences in cognitive style affect the type of information to be chosen, verbal or visual, for guiding navigation (Pazzaglia and De Beni 2001) and good imagery abilities are crucial to this aim. In this study, we considered regions involved in scene recognition and we studied whether the imagery ability could be associated with the intrinsic connectivity among the selected regions. We found that the outgoing connectivity in the right hemisphere regions were not related to the VVIQ (apart from the connectivity from OPA to PPA that negatively correlates with VVIQ score). We suppose that the right hemisphere could have different connectivity with the more medial regions, such as the hippocampus, or frontal regions during imagery of places. Previously, Boccia et al. (2017b) using psychophysiological interaction (PPI) analysis, found that right PPA showed higher connectivity with right RSC during perception and higher connectivity with the right HC during imagery of familiar landmarks.

We are aware that imagery involves a wide set of regions so that more studies are required to investigate other aspects of imagery abilities. Moreover, further research is needed to investigate the effect of scene-selective regions on the hippocampus, in task-based as well as resting-state designs. For this reason, there are some limitations in our study which can be addressed in future. First, we used volume-based ROIs centred on the local maximum of the region that was nearest the maxima in the group mean map as criteria used to choose scene-selective regions. A surface-based approach would better define regions that are functionally defined. Moreover, we analysed data from subjects during resting-state fMRI so that our results show the intrinsic interactions among scene-selective regions. We are aware that results from task-based effective connectivity among scene-selective regions might vary from results we found in this study. In our case, dynamic causal modelling for resting state is based on cross-spectral density and has no experimental design regressors, while results on task-based DCM strictly depend on the type of stimulus used and the task performed.

## Conclusions

In conclusion, our results provide new information about the causal interaction among brain regions involved in the perception and the imagery of scene/place and demonstrate that intrinsic fluctuations of signals among scene-selective regions reflect individual imagery abilities. Generally, understanding the complexity of the reciprocal influences among human brain regions is one of the most challenging goals for future neuroscientists. In our study, we decided to focus on regions belonging to the navigational system providing new insight in understanding how these regions communicate with each other. At the same time, since navigation is also dependent on imagery ability, we decided to test the relation of effective connectivity results with a behavioural measure. Our results confirm that the intrinsic connectivity among regions could be a key to explain the individual differences in navigational and possibly in other abilities.

## Supplementary Information

Below is the link to the electronic supplementary material.

Supplementary file1 (PDF 236 KB)

## Declarations

### Availability of data and material

Data are available upon request to the corresponding author in compliance with the institutional ethics approval.

### Code availability

Scripts used for running experiments and data analyses are included in SPM12.
