# Supplementary material for: Individual differences in mental imagery modulate effective connectivity of scene-selective regions during resting state
Source: Brain Struct Funct. 2022 Mar 21;227(5):1831–42. doi: 10.1007/s00429-022-02475-0 (PMC9098601; doi:10.1007/s00429-022-02475-0)
Supplement: Supplementary file 1 — Supplementary file1 (PDF 236 KB) [file 429_2022_2475_MOESM1_ESM.pdf]

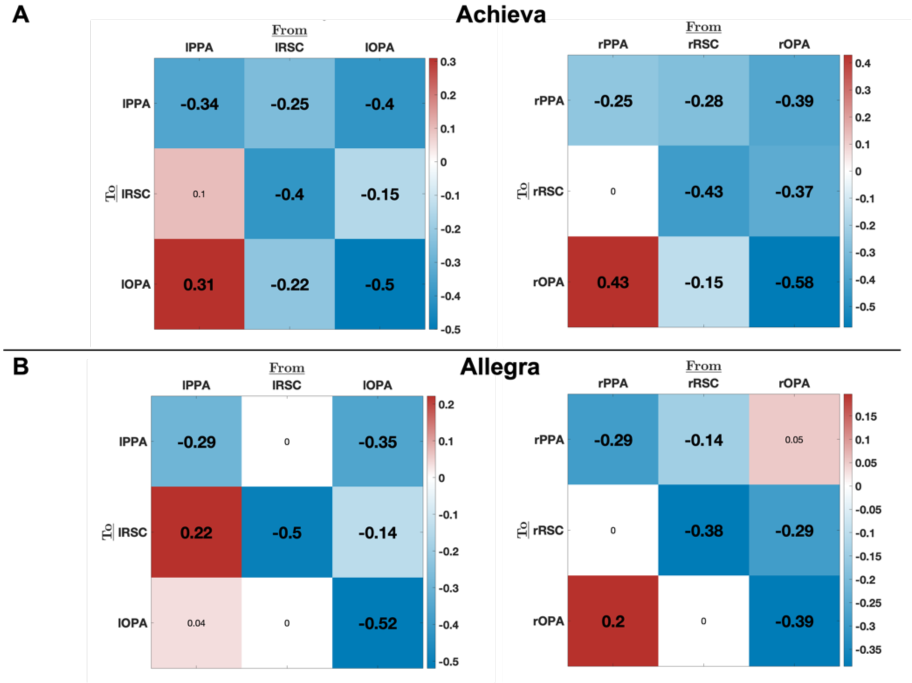

Supplementary Figure1: Parametric Empirical Bayes (PEB) analyses for data acquired using Achieva scanner (Panel A) and Allegra scanner (Panel B) respectively. Results of the left hemisphere are shown on the left part of the image whereas the results of the right hemisphere are shown on the right. Parameters with posterior probability higher than 95% are shown and are marked in bold type. Non suprathreshold parameters values are also reported in non-bold types in matrices. Connection strengths are displayed from pale red to dark red (i.e., excitatory), and from pale blue to blue (i.e., inhibitory). Data were collected on a Siemens Allegra 3T scanner for 18 of the 42 participants and on a Philips Achieva 3T scanner for the remaining 24 subjects. We controlled for the potential confound of using two different scanners by performing, at the second-level, two Parametric Empirical Bayes (PEB) analyses– conducted separately for each dataset. The two PEB analyses revealed comparable results between the scanners even though some differences in connectivity estimates occurred. For instance, in Achieva scanner (Supplementary Figure 1A) the outgoing connectivity from the left RSC to left PPA and from the left RSC to the left OPA was inhibitory (from left RSC to left PPA: connection strength = -0.25, posterior probability = 0.99; from left RSC to left OPA: connection strength = -0.22, posterior probability = 1.00) whereas in Allegra scanner (Supplementary Figure 1B) the outgoing connectivity between the same couplings didn't exceed the threshold and it was equal to 0.

A similar pattern is shown in the right hemisphere: the outgoing connectivity from RSC to OPA and from OPA to PPA couplings exceeded the threshold in Achieva scanner (Supplementary Figure 1A) but not in the Allegra scanner (Supplementary Figure 1B). Indeed, in Achieva scanner (Supplementary Figure 1A) the outgoing connectivity from the right RSC to right OPA was inhibitory (connection strength = -0.15, posterior probability = 0.99) whereas in Allegra scanner (Supplementary Figure 1B) the outgoing connectivity between the same couplings didn't exceed the threshold and it was equal to 0. Also, in Achieva scanner, the right OPA inhibited the right PPA (connection strength = -0.39, posterior probability = 1.00) whereas the same coupling didn't exceed the threshold (connection strength = 0.05, posterior probability =0.62) in Allegra scanner (Supplementary Figure 1B).

PEB analysis using small sample sizes can be sensitive to differences in subject-specific DCM estimates. In the present work, the scanner-specific samples might not have enough subjects to give us precise and robust connectivity estimates (e.g., because the priors have too much influence on the estimates). Hence, connections might change because of incomplete sampling and may be underestimated (i.e., because the prior mean is zero).
